# From Static to Dynamic: Smart Materials Pioneering Additive Manufacturing in Regenerative Medicine

**DOI:** 10.3390/ijms242115748

**Published:** 2023-10-30

**Authors:** Antreas Kantaros, Theodore Ganetsos

**Affiliations:** Department of Industrial Design and Production Engineering, University of West Attica, 12244 Athens, Greece

**Keywords:** regenerative medicine, smart materials, biomimetic, tissue engineering, dynamic constructs, shape memory polymers, biomedical engineering, 4D printing, 3D printing, biofabrication, dynamic biomaterials, tissue mimicry

## Abstract

The emerging field of regenerative medicine holds immense promise for addressing complex tissue and organ regeneration challenges. Central to its advancement is the evolution of additive manufacturing techniques, which have transcended static constructs to embrace dynamic, biomimetic solutions. This manuscript explores the pivotal role of smart materials in this transformative journey, where materials are endowed with dynamic responsiveness to biological cues and environmental changes. By delving into the innovative integration of smart materials, such as shape memory polymers and stimulus-responsive hydrogels, into additive manufacturing processes, this research illuminates the potential to engineer tissue constructs with unparalleled biomimicry. From dynamically adapting scaffolds that mimic the mechanical behavior of native tissues to drug delivery systems that respond to physiological cues, the convergence of smart materials and additive manufacturing heralds a new era in regenerative medicine. This manuscript presents an insightful overview of recent advancements, challenges, and future prospects, underscoring the pivotal role of smart materials as pioneers in shaping the dynamic landscape of regenerative medicine and heralding a future where tissue engineering is propelled beyond static constructs towards biomimetic, responsive, and regenerative solutions.

## 1. Introduction

Additive manufacturing, often referred to as 3D printing, has revolutionized the world of manufacturing and design over the past few decades. It represents a shift from traditional subtractive manufacturing processes, where materials are cut away to create a final product. Instead, additive manufacturing builds objects layer by layer, offering unprecedented design freedom and efficiency. However, the evolution of this technology has not stopped at three dimensions; it has taken a dynamic leap forward with the advent of 4D printing [1].

Three-dimensional printing, in its initial stages, allowed for the creation of complex and customized objects, from intricate prototypes to aerospace components. It gained popularity for its ability to reduce material waste, decrease lead times, and facilitate rapid prototyping. Yet, the true breakthrough came when researchers began to explore how materials could not only be added layer by layer but also programmed to change shape or properties over time, giving rise to the concept of 4D printing [2].

In 4D printing, the fourth dimension is time, which is integrated into the printed object’s functionality. This innovation relies on smart materials, such as shape memory polymers and stimulus-responsive materials, that react to external stimuli like heat, moisture, or light. By harnessing these responsive materials, 4D printing enables the creation of self-transforming structures, adaptive tools, and even biomedical devices [3].

One of the most remarkable applications of 4D printing lies in the realm of self-assembly. Objects printed with smart materials can respond to environmental changes autonomously, assembling themselves into a predetermined shape or structure without human intervention. This has profound implications for industries ranging from construction to space exploration, where the deployment of complex structures in remote or hazardous environments could be streamlined [4].

The convergence of 4D printing and regenerative medicine represents a significant amalgamation of technological expertise and advancements in healthcare. The utilization of 3D printing has significantly advanced the fields of medical device manufacture and tissue engineering. However, the emergence of 4D printing adds a new and innovative dimension to the field of regenerative medicine. In essence, the concept of 4D printing enhances the traditional three-dimensional structures generated through 3D printing by introducing the dimension of time. The temporal dimension is established by employing smart materials that respond to external stimuli, including temperature, humidity, and light [5]. The aforementioned materials possess the ability to react to their environment, hence facilitating changes in shape, self-organization, or other forms of dynamic activity inside the printed structures. Consequently, these materials exhibit the capacity to adapt and undergo evolutionary processes as time progresses [5].

At its essence, 4D printing involves the incorporation of intelligent materials capable of undergoing controlled and programmed changes in shape or alterations in properties when activated by specific stimuli. These stimuli encompass a wide range of factors, including temperature changes, humidity levels, light intensity, and mechanical forces. The materials utilized in 4D printing exhibit an extraordinary capacity to react to external stimuli, so commencing a process of transformation that results in dynamic and adaptive behavior [6].

The underlying principle of 4D printing is the precise design and fabrication of intelligent materials possessing the desired shape-altering properties. These materials can be categorized into two primary classifications: shape memory materials and stimulus-responsive materials [7]. Shape memory materials possess the inherent ability to “recall” and revert back to a predetermined shape when triggered by an external stimulus. Stimulus-responsive materials, on the other hand, have the ability to undergo changes in response to external stimuli. Reversible modifications in their properties or shape occur when they are subjected to specific triggers [8]. In order to fully harness the capabilities of 4D printing, it is imperative to adopt a multidisciplinary approach. Researchers and engineers utilize knowledge from various disciplines, including materials science, mechanical engineering, computer science, and design, in order to provide novel approaches for material selection, design optimization, and fabrication techniques. Computational modeling and simulation are essential in forecasting and enhancing the performance of 4D-printed structures, facilitating precise manipulation of the intended shape transformations [9].

Smart materials, including shape memory polymers (SMPs), liquid crystal elastomers (LCEs), magnetic shape memory alloys (MSMAs), biodegradable polymers, and cell-laden materials, have emerged as key raw materials in the realm of additive manufacturing, particularly in the context of 4D printing, for advancing regenerative medicine applications. In 4D printing, these materials demonstrate their transformative potential by not only enabling the creation of complex, three-dimensional structures but also imbuing them with the ability to change shape or function over time in response to environmental stimuli. SMPs, for instance, can be 3D-printed into temporary implants that regain their pre-programmed shape when exposed to body heat or other triggers, making them ideal for creating customizable, patient-specific scaffolds and implants for tissue regeneration [9].

For example, LCEs, with their inherent anisotropic properties and responsiveness to external stimuli like heat or light, have been harnessed to print intricate structures that mimic the mechanical behavior of natural tissues. This capability is crucial for designing tissue constructs with tunable mechanical properties that closely match the target tissue, facilitating better integration and functionality in regenerative medicine [10].

Also, MSMAs offer a unique opportunity in 4D printing as they can be incorporated into implants or devices that change shape under the influence of a magnetic field. This property can be exploited to remotely control the shape and function of implants within the body, enabling dynamic adjustments and controlled drug release for regenerative purposes [11].

In addition, biodegradable polymers, a staple in 3D and 4D printing, allow for the fabrication of temporary supports, drug delivery systems, or even biodegradable scaffolds that gradually degrade as new tissue forms, eliminating the need for secondary surgeries to remove implants [12,13].

Moreover, the integration of cell-laden materials into 4D printing processes holds tremendous potential. By precisely depositing cells along with smart materials, it becomes possible to create highly functional and biomimetic tissue constructs that can evolve and adapt over time, closely mimicking the natural regenerative processes of the human body [14,15].

In this context, 4D printing unveils a range of promising applications. More specifically, tissue scaffolds have long played a critical role in providing structural support and a conducive environment for the complex process of tissue regeneration. Traditional 3D-printed tissue scaffolds have been pivotal, offering a foundation for cells to adhere, proliferate, and develop into functional tissues [16]. However, the evolving landscape of regenerative medicine demands more advanced solutions, and this is where the transformative potential of 4D printing comes into play. Four-dimensionally printed tissue scaffolds, crafted from smart materials with responsive properties, represent a profound evolution [17]. They transcend the static nature of 3D printing, possessing the remarkable ability to mimic the dynamic characteristics of native tissues. These scaffolds can adapt, changing shape, mechanical properties, and even biochemical composition in response to signals from surrounding cells or the evolving tissue microenvironment [18]. This inherent responsiveness creates an exceptionally biomimetic environment, closely mirroring the dynamic interplay of forces and signals found in natural tissue growth and repair processes [19]. Cells within this finely tuned environment thrive, proliferate, and differentiate with enhanced efficiency, accelerating the regenerative process and yielding tissues that closely resemble their natural counterparts in terms of structure, function, and resilience [20,21,22].

On the other hand, 4D-printed drug delivery systems mark a pioneering advancement in the field of medical therapeutics. These ingenious systems have the capability to release therapeutic agents with unparalleled precision, responding dynamically to specific physiological changes within the body. Consider, for instance, a 4D-printed implant meticulously designed to release growth factors or medications in response to nuanced shifts in parameters such as pH, temperature, or other relevant markers at the precise target tissue site [23]. This level of exquisite control over drug release not only enhances the efficacy of regenerative therapies but also minimizes potential side effects and optimizes therapeutic outcomes. Also, in the field of self-assembling constructs, smart materials in 4D printing can enable the creation of self-assembling tissue constructs. These constructs can autonomously transform from a compact state suitable for minimally invasive delivery to a functional, three-dimensional tissue structure once inside the body. This minimizes surgical invasiveness and accelerates tissue repair [24].

In addition, in the field of patient-specific implants, 4D printing allows for the customization of implants to match the unique anatomy of each patient. These implants can adapt and conform to the patient’s body over time, reducing the risk of complications and improving long-term outcomes. What is more, by closely mimicking the dynamic behavior of living tissues, 4D-printed constructs can encourage cell proliferation, differentiation, and tissue remodeling. This level of biological mimicry is a significant step toward achieving successful tissue regeneration [25].

In conclusion, smart materials play a prominent role in the advancement of additive manufacturing and 4D printing in the field of regenerative medicine. The capacity to integrate accuracy, customization and adaptable functionality inside printed structures presents novel opportunities for tissue engineering, medication administration, and individualized therapies, thereby enhancing the potential of regenerative medicine.

## 2. Smart Materials

### 2.1. Characteristics of Shape Memory Polymers (SMPs) 

**Figure 1:**
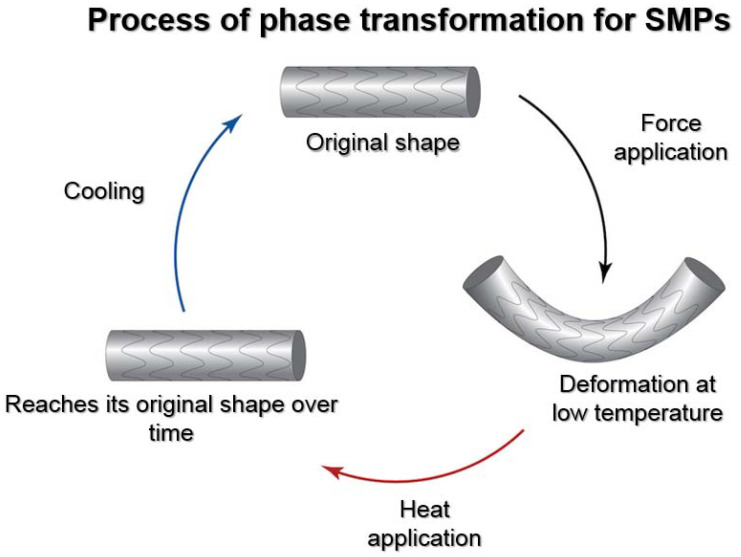
Phase transformation process for SMPs.

Shape memory polymers (SMPs) are a captivating category of biomimetic intelligent materials that exhibit the capability to retain their initial shape and recover it following deformation, prompted by specific stimuli such as temperature, light, or pH alterations. SMPs are commonly comprised of a polymer network that possesses the potential to manifest two unique states: a temporary shape that can be easily manipulated and a permanent shape that the material will revert back to upon activation [25]. The distinctive behavior observed in this context arises from the reversible transitions occurring between the glassy and rubbery states of the material, facilitating substantial deformation and shape recovery [26]. Figure 1 depicts the phase transformation process for SMPs.

For example, shape flexibility is a notable characteristic for which SMPs are widely recognized. This inherent attribute enables them to undergo shape alteration in response to external stimuli, like temperature, light, or pH. The versatility demonstrated in the realm of regenerative medicine holds immense value. SMPs possess the capability to undergo a transition from a compact form, which facilitates minimally invasive delivery to the desired location within the body, to a predetermined functional shape that offers ideal support and prompts tissue regeneration. The aforementioned dynamic response facilitates a harmonious integration with the patient’s anatomical structure, hence reducing any potential discomfort and enhancing the efficacy of regenerative treatments [27].

The importance of biocompatibility in the field of regenerative medicine cannot be overstated, as it directly influences the success of therapeutic interventions. SMPs possess the advantageous characteristic of being customizable to exhibit a high degree of compatibility with biological systems. These materials can be intentionally designed to demonstrate reduced levels of toxicity and inflammation while simultaneously promoting the attachment and growth of cells. The process of customisation guarantees the seamless integration of SMP-based constructions with host tissues, hence minimizing the potential for negative reactions and facilitating the regeneration of tissue [28].

The integration of SMPs with 4D printing techniques has brought substantial advancements in the field of regenerative medicine. When combined with 4D printing, which involves the production of objects that can transform their shape over time, SMPs offer remarkable possibilities for creating dynamic, adaptable structures in regenerative medicine.

For example, it enables the advancement of precise medication delivery systems. SMP-based carriers have the ability to systematically release bioactive substances, growth factors, or pharmaceuticals in a regulated manner [29]. The precise control of drug release enables the manipulation of cellular responses during the process of tissue repair and regeneration, hence improving the effectiveness of therapeutic interventions. In the context of bone regeneration, it has been observed that scaffolds based on SMPs had the ability to selectively release bone growth factors throughout distinct phases of the healing process, hence expediting the development of new bone tissue [30].

SMPs utilized in the context of 4D printing have the capability to facilitate the development of regenerative treatments that are tailored to the specific needs of individual patients. Clinicians have the ability to construct individualized implants and scaffolds by integrating patient data, including medical imaging and genetic information, into the design process. The implementation of patient-centric solutions aims to optimize compatibility and efficacy through the customization of treatment based on the individual patient’s distinct anatomical and biological attributes [31].

In another instance, the combination of SMPs and 4D printing allows the creation of dynamic tissue scaffolds that can mimic the mechanical properties of natural tissues. These scaffolds can support cell growth and tissue regeneration in a way that closely resembles the natural environment. By incorporating biochemical cues and the ability to change shape, they can guide tissue regeneration more effectively [32].

Also, SMPs integrated with 4D printing techniques can be used to create adaptive orthopedic devices that respond to the body’s movement and changes in biomechanical forces. For instance, 4D-printed orthopedic implants made from SMPs can adjust their shape to support bone regeneration and improve the healing process, thereby reducing the risk of implant failure and enhancing patient outcomes [33].

**Figure 2:**
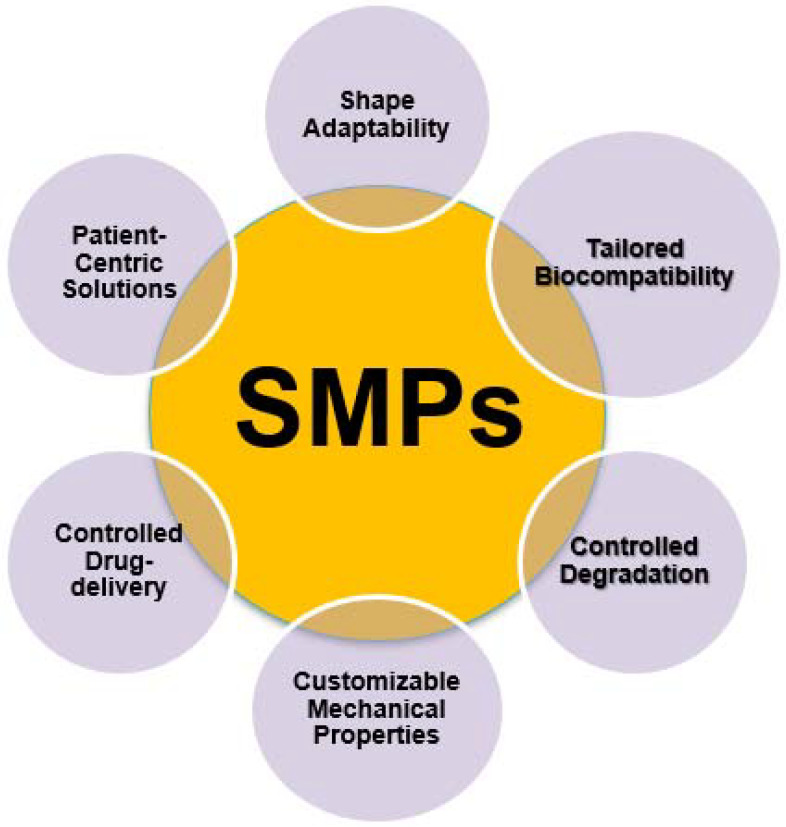
Characteristics of shape memory polymers (SMPs) in the biomedical field.

In addition, in the case of vascular stents SMP-based 4D-printed vascular stents offer the advantage of dynamically adapting to changes in blood flow and vessel structure. These stents can promote better integration with the surrounding tissue and reduce the risk of complications, such as restenosis or thrombosis, thus improving the long-term efficacy of the treatment [34]. Figure 2 depicts the characteristics of shape memory polymers (SMPs) in the biomedical field.

### 2.2. Characteristics of Liquid Crystal Elastomers (LCEs) 

**Figure 3:**
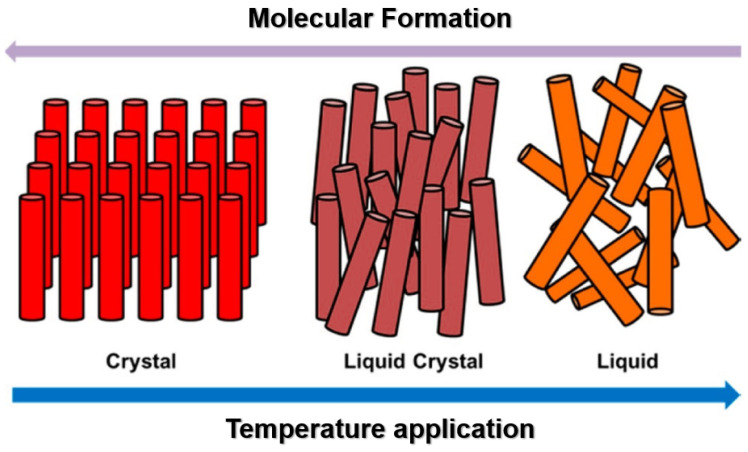
Phase transformation process for LCEs.

Liquid crystal elastomers (LCEs) represent a class of advanced materials with immense potential in the field of regenerative medicine. LCEs are unique compounds known for their ability to exhibit both liquid-like and solid-like properties simultaneously. These materials are composed of polymer chains with liquid crystal components, allowing them to undergo substantial shape changes in response to external stimuli, such as heat or light. This exceptional property makes LCEs valuable raw materials for various applications in regenerative medicine [35]. Figure 3 depicts the phase transformation process for LCEs.

One of the most exciting aspects of LCEs is their adaptability to the dynamic needs of regenerative medicine. Unlike traditional materials, LCEs can mimic the mechanical properties of natural tissues, making them ideal candidates for tissue engineering scaffolds. By precisely tailoring the chemical composition and molecular structure of LCEs, scientists can create scaffolds that not only support cell growth and tissue regeneration but also respond to the mechanical cues required for proper tissue development [36].

Furthermore, the controlled release of therapeutic agents is a crucial aspect of regenerative medicine. LCEs’ responsiveness to external stimuli, such as temperature changes, enables them to act as smart carriers for drug delivery systems. This capability ensures that regenerative therapies can be delivered precisely to the target site, promoting tissue repair while minimizing side effects and improving patient outcomes [37,38].

In addition to tissue engineering and drug delivery, LCEs hold promise in the development of smart implants that can adapt to the ever-changing conditions within the human body. These materials can be integrated into prosthetics or implants, offering patients enhanced mobility and comfort, particularly in cases where the implant must interact with living tissue [39].

More specifically, LCEs are poised to revolutionize the field of tissue engineering by offering a dynamic and adaptive platform for creating highly specialized scaffolds. Unlike conventional materials, LCE-based scaffolds possess the unique ability to alter their mechanical properties in response to external stimuli. This feature allows researchers to design scaffolds that not only provide structural support but also actively engage with surrounding cells and tissues [40]. LCE scaffolds can be engineered to respond to factors such as temperature, light, or pH levels, enabling precise control over the microenvironment for tissue growth and regeneration. This adaptability is particularly valuable when attempting to replicate the complex mechanical properties of native tissues [41]. For example, in orthopedic applications, LCE scaffolds can mimic the resilience of cartilage, adapting their stiffness and flexibility to match the natural tissue. In cardiac tissue engineering, LCEs can simulate the contractile properties of heart muscle, fostering functional tissue growth. Moreover, LCE-based scaffolds can actively respond to changes in tissue stress, helping to guide cell behavior [42]. When cells exert mechanical forces on the scaffold, LCEs can adjust their mechanical properties accordingly, signaling to cells to differentiate, proliferate, or produce extracellular matrix components as needed. This capability allows for more sophisticated and biologically relevant tissue engineering approaches, where the scaffold becomes an active participant in tissue development [43].

Also, LCEs offer an innovative approach to drug delivery systems in regenerative medicine. Their remarkable ability to respond to external stimuli, such as temperature changes or mechanical stress, makes them well-suited for precisely controlled drug release, significantly enhancing the efficacy of regenerative therapies. LCEs can be tailored to encapsulate a wide range of therapeutic agents, including growth factors, stem cells, and small-molecule drugs. These agents can be released from LCE matrices in response to specific triggers, ensuring that they are delivered directly to the target tissue or organ at the right time and in the right amount [44]. This targeted drug delivery minimizes potential side effects associated with systemic drug administration and maximizes therapeutic benefits.

Moreover, LCEs can respond to the unique microenvironment of the body, allowing for on-demand drug release. For instance, in tissue regeneration, LCE-based drug carriers can be designed to release growth factors when exposed to certain biochemical signals indicative of tissue damage or inflammation. This dynamic responsiveness ensures that regenerative processes are initiated precisely when needed, promoting more effective tissue repair and regeneration [44].

In addition to their controlled drug release capabilities, LCEs’ biocompatibility and adaptability make them ideal candidates for developing implantable drug delivery devices. These devices can be seamlessly integrated into the body, continuously monitoring and responding to changing physiological conditions. This approach holds particular promise in the treatment of chronic conditions, such as osteoarthritis or diabetes, where sustained, localized drug delivery can significantly improve patient outcomes. In this context, liquid crystal elastomers in drug delivery systems are set to play a pivotal role. Their ability to provide targeted and responsive therapy delivery promises to optimize regenerative treatments, offering patients more efficient and tailored solutions for tissue repair, organ regeneration, and overall improved health [45].

LCEs are, also, at the forefront of innovation in the creation of smart implants that have the ability to adapt to the dynamic and ever-changing conditions within the human body. These adaptive materials offer a remarkable solution to some of the challenges associated with traditional implants by providing a level of responsiveness and functionality previously unattainable. LCE-based implants can be designed to respond to various environmental cues, including changes in temperature, pH levels, or mechanical forces. For example, in orthopedics, LCE-based artificial joints or prosthetic limbs can adjust their stiffness and flexibility in response to the user’s movement and the surrounding tissue conditions. This adaptability ensures that the implant closely mimics the natural function of the replaced body part, leading to improved mobility and comfort for the patient [46].

Furthermore, LCEs are biocompatible materials, which means they are well-suited for integration with living tissues. This property is especially valuable when developing implantable devices for regenerative medicine. LCE-based implants can actively engage with surrounding tissues, promoting tissue integration and reducing the risk of complications such as implant rejection or inflammation. In the context of regenerative medicine, LCE-based implants can also facilitate the controlled release of therapeutic agents, enhancing the regeneration process. By incorporating growth factors or stem cells into the implant matrix, these devices can deliver regenerative signals directly to the surrounding tissues, accelerating the healing and regrowth of damaged or diseased areas [47].

The biocompatibility of LCEs is a crucial advantage in regenerative medicine. These materials are well-tolerated by the body, minimizing the risk of adverse reactions or immune responses when used as implantable devices or scaffolds. This compatibility enables seamless integration with the patient’s own tissues, fostering a more natural and efficient regenerative process. LCEs can be customized to mimic the specific mechanical characteristics of various tissues, including bone, cartilage, muscle, and even neural tissue. This tunability ensures that the scaffolds or implants made from LCEs closely resemble the natural tissue they are intended to replace or support. For example, in bone tissue engineering, LCE-based scaffolds can be designed to exhibit the right stiffness and porosity for bone regeneration, promoting healthy bone growth and integration [47].

**Figure 4:**
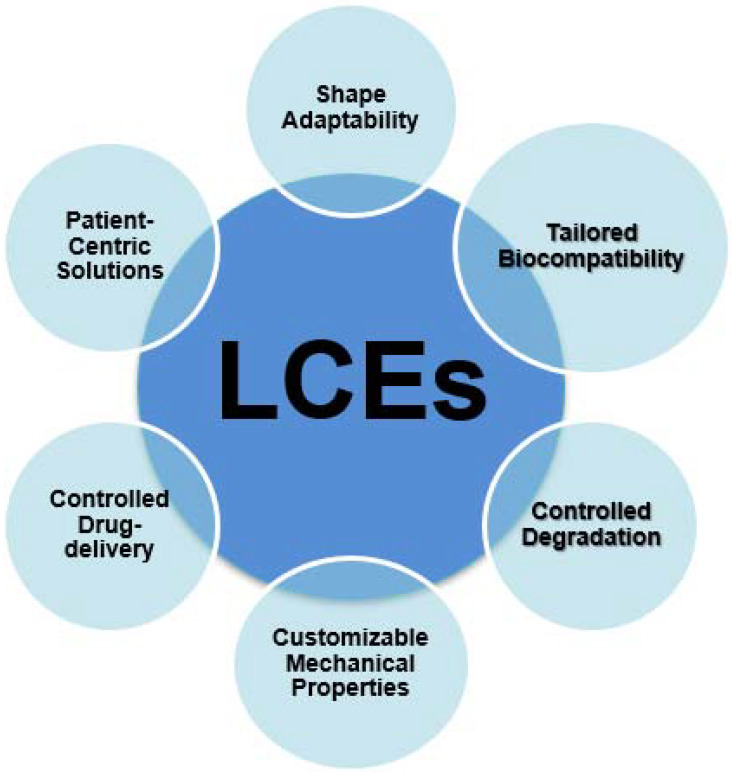
Characteristics of LCEs in the biomedical field.

Furthermore, LCEs can be tailored to respond to specific environmental cues within the body, such as temperature changes or variations in pH levels. This adaptability allows for dynamic adjustments in the mechanical properties of LCE-based materials, ensuring that they provide the necessary support and signals for tissue growth and regeneration as conditions evolve [47]. Figure 4 depicts the characteristics of LCEs in the biomedical field.

### 2.3. Characteristics of Magnetic Shape Memory Alloys (MSMAs) 

**Figure 5:**
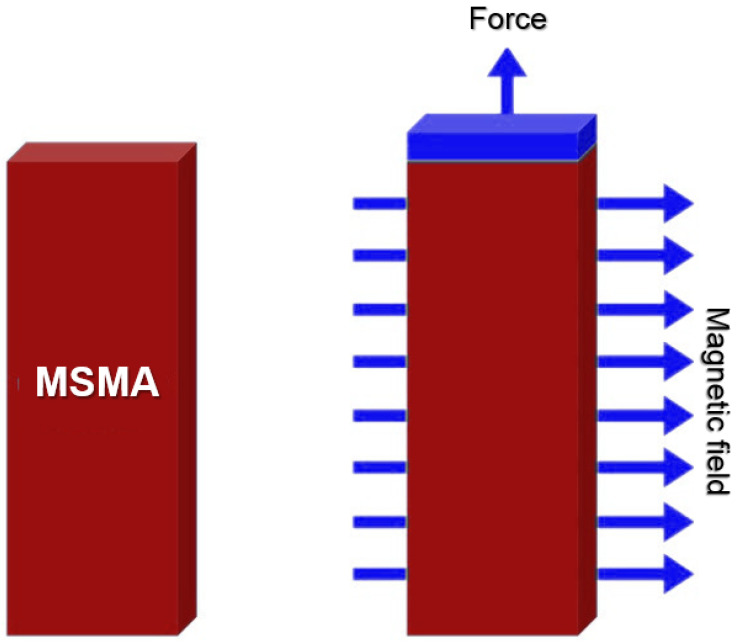
Schematic representation of MSMA modus operandi.

Shape memory alloys (SMAs) have emerged as a significant innovation in the dynamic field of medical technology. Their exceptional capacity to retain and revert to a predetermined shape when exposed to specific stimuli has revolutionized multiple sectors. Among the most promising applications of SMAs is their integration into the domain of 4D printing for medical implants and devices. Shape memory alloys, often referred to as SMAs or MSMAs (magnetic shape memory alloys), are materials that possess a unique property: they can undergo a reversible transformation in shape when exposed to external triggers such as heat, stress, or, in the case of MSMAs, a magnetic field. This distinctive attribute makes them ideal candidates for use in 4D printing [48]. Figure 5 shows a schematic representation of MSMA modus operandi.

One of the primary benefits of Magnetic Shape Memory Alloys (MSMAs) in the field of 4D printing for medical implants and devices lies in their remarkable biocompatibility. The distinctive characteristic of MSMAs guarantees their successful integration into the human body, as they do not elicit any unfavorable reactions or immune responses. The compatibility of shape memory alloys (SMAs) with biological tissues and fluids renders them a very suitable option for the fabrication of implants and devices, as it effectively reduces the likelihood of difficulties [49]. Furthermore, the presence of this biocompatibility factor creates an opportunity for the advancement of future medical innovations that possess the ability to not only alter their shape in response to a magnetic field but also seamlessly integrate with the complex biological systems of the human body. This holds the potential to enhance the safety and efficacy of medical interventions [50].

One notable advantage of MSMAs when used as raw material in 4D printing operations is its exceptional capacity to provide a high degree of precise control. The utilization of a magnetic field to induce shape changes in MSMAs enables precise and regulated transformations, which is of utmost importance in medical applications that require a high degree of accuracy. The precise nature of MSMAs guarantees that implants or devices made from them can effectively and accurately react to particular stimuli, so achieving ideal outcomes while limiting the likelihood of errors or consequences. The capacity to precisely adjust the characteristics of these materials presents a multitude of opportunities for developing medicinal remedies customized to the distinct requirements of individual patients, hence enhancing the prospects of more efficient and personalized healthcare interventions [51].

In addition, the incorporation of MSMAs into the process of 4D printing not only contributes to advancements in the domain of medical implants and equipment, but also facilitates a heightened degree of personalization in healthcare applications. Metallic shape memory alloys (MSMAs) have the capability to facilitate the development of customized implants and devices that possess the ability to conform to the unique needs of individual patients. The degree of customization involved in designing medical procedures can significantly influence treatment outcomes by effectively addressing the specific needs of each individual, rather than adopting a generic approach. The utilization of MSMAs in 4D printing offers significant potential for customisation in various healthcare applications, such as orthopedic implants tailored to individual anatomies or stents capable of adapting to fluctuations in blood flow. This customization capability holds promise for enhancing the effectiveness of healthcare solutions, prioritizing patient-centric approaches, and eventually leading to improved patient comfort and treatment outcomes [52].

**Figure 6:**
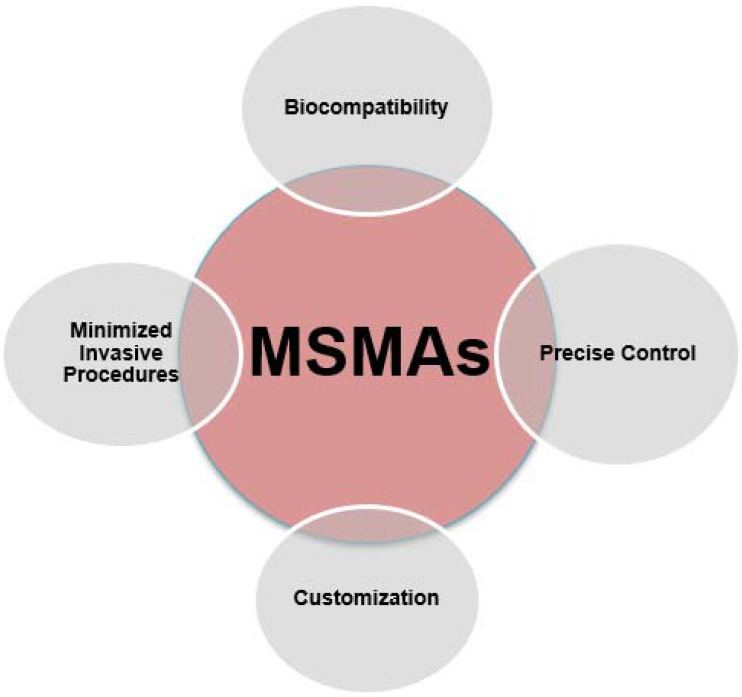
Characteristics of magnetic shape memory alloys (MSMAs) in the biomedical field.

Moreover, the utilization of MSMAs in the context of 4D printing holds promise for substantial mitigation of the necessity for intrusive medical interventions. Devices and implants fabricated using shape memory alloys (SMAs) have the ability to dynamically adjust and alter their form in accordance with physiological variations or environmental inputs. For example, stents that possess shape-changing capabilities through the integration of MSMAs (shape memory alloys) can effectively adapt to changes in blood flow, hence enhancing their functionality and reducing the likelihood of adverse events such as restenosis or thrombus formation [53]. The enhanced adaptability of this technique not only contributes to improved patient comfort but also diminishes the need for subsequent surgical procedures or interventions, hence advocating for a healthcare approach that is less intrusive and more patient-centered. The utilization of minimally invasive surgical procedures, commonly referred to as MSMAs, has significant importance in the progression of medical technology. This advancement offers the potential for a reduction in invasive interventions, which in turn contributes to enhanced patient outcomes and overall quality of life [53]. Figure 6 depicts the characteristics of magnetic shape memory alloys (MSMAs) in the biomedical field.

### 2.4. Characteristics of Biodegradable Polymers 

The emergence of biodegradable polymers has introduced a range of opportunities in the field of 3D and 4D printing technologies within the dynamic realm of contemporary medicine. These polymers have gained recognition for their environmentally benign characteristics and have been integral in numerous healthcare applications. They enable the creation of temporary supports, drug delivery systems, and particularly noteworthy biodegradable scaffolds that progressively decompose as new tissue develops. This innovative advancement not only mitigates the ecological impact of medical procedures but also obviates the necessity for subsequent surgical interventions to extract implants, signifying a significant transformation in patient healthcare and recuperation [54]. This combination offers numerous advantages and potential applications in the field. Recent advancements, however, have been met with specific challenges that need to be addressed to fully realize the potential of this technology.

For example, in the case of tailored tissue scaffolds, researchers have made significant progress in developing biodegradable polymer-based 4D-printed tissue scaffolds that can mimic the mechanical and structural properties of natural tissues. These scaffolds can provide a temporary support structure for tissue regeneration, gradually degrading as the tissue grows, ensuring minimal foreign material remains in the body [55]. Also, recent advancements involve the development of 4D-printed biodegradable polymer-based cell encapsulation systems that can protect transplanted cells, providing a controlled microenvironment for their growth and integration within the host tissue. These systems enable the sustained release of growth factors, promoting enhanced tissue regeneration and repair. In addition, integrating biodegradable polymers with 4D printing has led to the creation of intricate drug delivery devices capable of controlled and targeted drug release. These devices can be designed to degrade over time, ensuring the gradual and sustained delivery of therapeutic agents to the specific site of interest, thereby enhancing the efficacy of regenerative treatments [56].

Challenges that arise from the use of biodegradable polymers in the field of 3D and 4D printing technology have to do with material properties and stability where ensuring the structural integrity and mechanical stability of biodegradable polymers during the printing process and throughout the degradation period remains a significant challenge. Researchers need to balance the degradation rate with the required mechanical properties to ensure the longevity of the printed constructs. Also, regarding biocompatibility and toxicity factors, while biodegradable polymers are generally considered biocompatible, the degradation by-products may raise concerns regarding their potential toxicity and impact on the surrounding tissue. Further studies are needed to ensure the complete biocompatibility and safety of these materials and their degradation products. It should also be mentioned that achieving high-resolution, complex structures with biodegradable polymers using 4D printing techniques can be challenging. Improving the printing resolution and accuracy is crucial to fabricate intricate structures that closely mimic the complex architecture of native tissues and organs [57].

**Figure 7:**
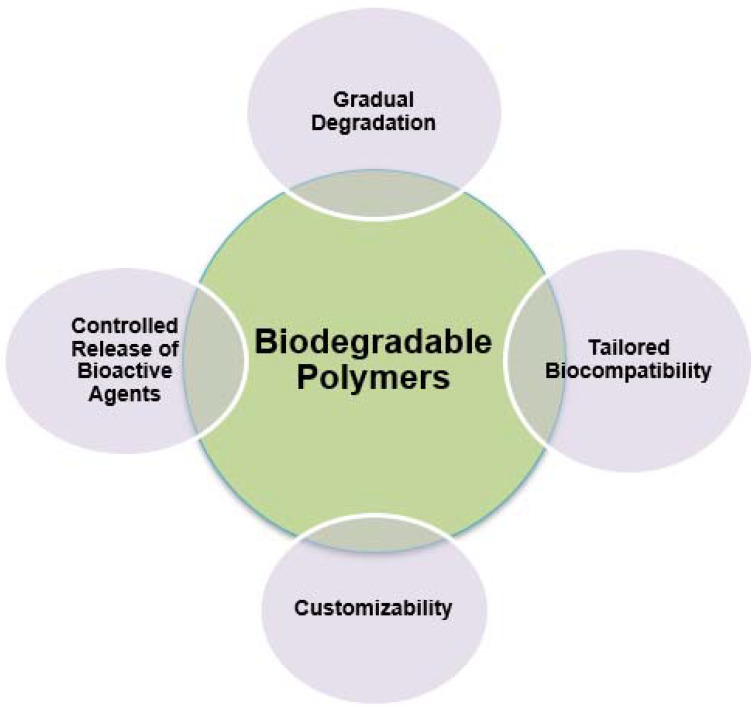
Characteristics of biodegradable polymers in the biomedical field.

On the other hand, prospects arising from the use of biodegradable polymers in the field of 3D and 4D printing technology have to do with personalized implants and prosthetics, where the integration of biodegradable polymers with 4D printing holds the promise of producing personalized implants and prosthetics that can adapt to the dynamic needs of individual patients, thereby improving their functionality and biocompatibility. Moreover, regarding bioresorbable vascular stents, biodegradable polymer-based 4D-printed vascular stents offer the potential to address the limitations associated with traditional metallic stents, providing temporary support to the blood vessels during the healing process before gradually degrading, thus reducing the risk of long-term complications. Also, as far as organ-on-a-chip models are concerned, integrating biodegradable polymers with 4D printing techniques can facilitate the development of advanced organ-on-a-chip models that closely mimic the physiological environment of human organs, enabling more accurate preclinical studies and drug testing [57]. Figure 7 depicts the characteristics of biodegradable polymers in the biomedical field.

### 2.5. Characteristics of Cell-Laden Materials 

The incorporation of cell-laden materials into the domain of 4D printing signifies a significant paradigm shift in the discipline of regenerative medicine, signaling the emergence of a new age. This innovative methodology enables scientists, researchers, and medical professionals to create dynamic, living structures that exhibit the extraordinary capacity to adapt and evolve through time [58]. These new materials represent a significant departure from static, conventional structures as they incorporate the vital presence of cells inside their matrix, thus signaling a paradigmatic transformation. The integration of biological and technological elements is responsible for endowing cell-laden materials with a distinct array of attributes, so conferring upon them significant use within the expansive domain of tissue engineering and regenerative medicine applications [58].

Cell-laden materials play a fundamental role in the field of regenerative medicine owing to their intrinsic biological functionality. The aforementioned materials serve as containment structures for living cells, which act as active agents within the constructs and play a vital role in the process of regeneration. The biological activity of these entities include their capacity to perceive and react to the surrounding microenvironment [59]. By means of complex signaling pathways and intercellular communication, these cells possess the ability to effectively coordinate with adjacent cells, so orchestrating a harmonious interplay of chemical cues that facilitate the process of tissue regeneration. The intricate network of contacts, regulated by the encapsulated cells, plays a crucial role in directing the development of functional and long-lasting tissue structures. The bioactivity of cell-laden materials is a crucial aspect of regenerative medicine. The presence of living entities within these constructs significantly influences the process and outcome of tissue regeneration, resulting in the development of durable and resilient tissue structures [59].

The maintenance of cellular viability within these materials is of utmost significance in the field of regenerative medicine. Cell-laden constructions are specifically engineered to provide a conducive and supportive milieu that ensures the well-being and robustness of the enclosed cells. The implementation of protective measures is crucial throughout the 4D printing procedure, and this safeguarding persists while the structure assimilates into the recipient tissue. The preservation of cell viability is crucial to ensure the presence and optimal functionality of cells, enabling their active participation in the regenerative process through activities such as proliferation, differentiation, and secretion of important extracellular matrix components [60].

The diversity of cell-laden materials lies in their ability to accommodate various cell types particular to different tissues. Researchers and medical practitioners have the ability to carefully choose and include specific cell populations that closely correspond to the tissue being focused on for the purpose of regeneration. For example, in the context of cartilage regeneration, the incorporation of chondrocytes is commonly observed, but in the field of cardiac tissue engineering, the utilization of cardiomyocytes is often considered. The particularity of this specificity guarantees that the cells are prepared to regenerate tissue with identical biological properties as the original tissue, hence enhancing the probability of functional and seamless integration [61].

The scaffolds generated by the utilization of cell-laden materials in the process of 4D printing exhibit a high degree of customization. Researchers possess the liberty to exercise their creative abilities in designing the structural characteristics of these scaffolds, including pore dimensions, morphology, and overall arrangement, with the intention of closely emulating the properties of the original tissue. In addition, it is possible to customize the mechanical qualities, such as stiffness and elasticity, in order to align with the specific requirements of the tissue that is undergoing regeneration. The degree of customization involved in the scaffolds guarantees that they offer the most effective support and cues for the growth of tissues [62].

The distinguishing factor of 4D printing with cell-laden materials is in the dynamic reaction demonstrated by these structures when exposed to environmental stimuli. Fundamentally, these entities exhibit a degree of flexibility that enables them to undergo modifications in their form, characteristics, or functionalities over a period of time in reaction to shifting circumstances within the organism. The ability to respond promptly and effectively is of utmost importance in the field of regenerative medicine, especially when addressing the healing and regeneration of tissues that require adjustment to mechanical or pharmacological stimuli. This facilitates the active participation of these constructions in the repair of tissue function and structure.

Cell-laden materials possess the capability to be manipulated in order to achieve controlled and precise release of bioactive substances, including growth factors and signaling chemicals. The regulated release mechanism serves a crucial function in directing cellular activities within the constructs. As an illustration, it has the capacity to induce cellular proliferation in the initial phases of tissue regeneration and then transition towards facilitating tissue maturation. The degree of regulation exerted on biochemical signaling significantly amplifies the accuracy and efficacy of tissue engineering methodologies [63].

One of the practical advantages associated with the utilization of cell-laden materials with 4D printing techniques is the ability to mitigate the necessity for invasive subsequent surgical procedures. As these constructions undergo adaptation and evolution in response to their specific microenvironment, they effectively obviate the need for further treatments. The decrease in invasive procedures not only serves to minimize patient discomfort and recovery time but also contributes to the reduction in healthcare expenses and mitigates the dangers associated with undergoing several surgeries [64].

The utilization of cell-laden materials enables the implementation of regenerative treatments tailored to individual patients. The integration of a patient’s autologous cells or cells produced from their own tissues enables the development of individualized regenerative therapies. The aforementioned treatments optimize compatibility and efficacy by leveraging the patient’s own biological composition, hence augmenting the likelihood of successful tissue regeneration and integration [65].

**Figure 8:**
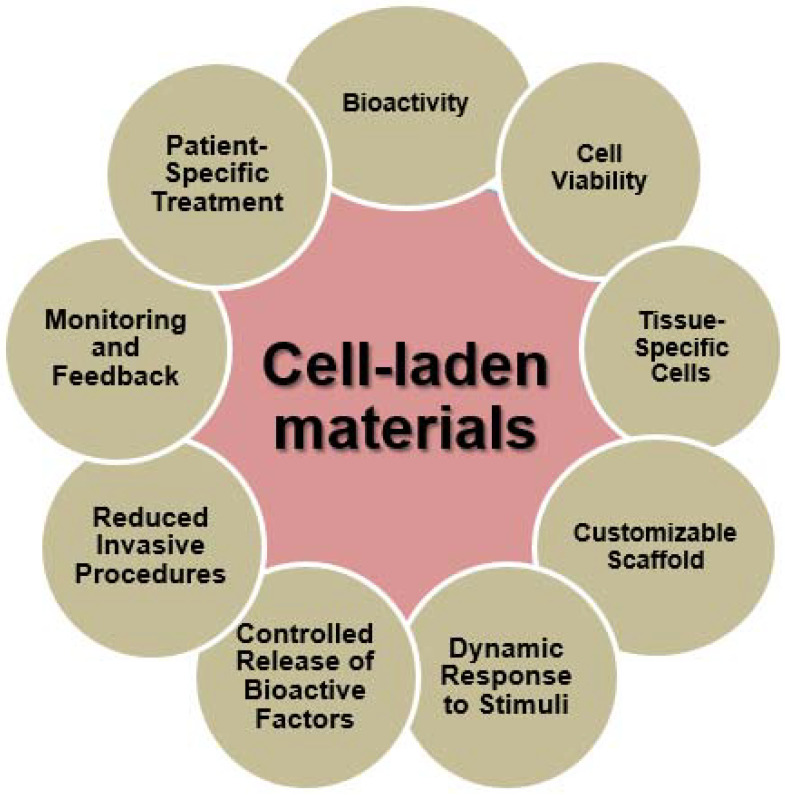
Characteristics of cell-laden materials in the biomedical field.

Cutting-edge 4D printing methodologies have the capacity to integrate sensors, imaging modalities, or alternative monitoring instruments in order to offer instantaneous feedback on the advancement of tissue regeneration within the constructs including cells. Continuous monitoring allows clinicians and researchers to evaluate the efficacy of the treatment, make prompt modifications, and acquire useful insights regarding the regenerative process. Figure 8 depicts the characteristics of cell-laden materials in the biomedical field [65].

## 3. Regenerative Medicine Applications of Smart Materials

### 3.1. SMPs Applications

SMPs represent a fascinating class of materials with a wide range of biomedical applications due to their unique ability to change shape in response to external stimuli. These versatile polymers have garnered significant attention in recent years for their potential to revolutionize various aspects of healthcare and medicine [66].

More specifically, in the field of invasive surgery, a field that has revolutionized medicine by reducing patient trauma, shortening recovery times, and improving surgical outcomes. SMPs are versatile materials used in the development of navigation and catheterization tools that can adapt to the intricate anatomical structures of the human body, such as cardiac catheters that respond to body temperature to navigate through blood vessels with precision. In tumor treatment, SMP-based probes and needles change shape upon reaching their target, ensuring controlled and targeted delivery of therapies while minimizing damage to healthy tissues. Endoscopic instruments integrated with SMPs enhance flexibility and adaptability during procedures, improving precision and patient comfort. SMPs also find use in suture materials and closure devices that respond to temperature changes, simplifying suturing, especially in challenging areas. Additionally, they can be employed for drug delivery during surgery, with SMP-based implants releasing medications gradually in response to body temperature, enhancing precision. In biopsy and tissue sampling devices, SMPs change shape to safely collect tissue samples, reducing invasiveness and improving diagnostic accuracy [66].

In addition, drug delivery systems employing SMPs represent a significant leap in personalized medicine and targeted therapy. These smart polymers respond to external stimuli, such as temperature or pH changes, with precision and reliability. When used in drug delivery, SMPs can encapsulate medications and release them gradually at the desired site within the body. This controlled and triggered drug release ensures that therapeutic agents reach their intended targets in a highly targeted manner. This not only increases the therapeutic efficacy but also minimizes potential side effects by reducing exposure to healthy tissues [67]. SMP-based drug delivery systems hold immense promise in treating various medical conditions, from chronic diseases to cancer, ushering in a new era of more efficient and patient-friendly treatments. As research continues to refine these systems, we can expect even more tailored and effective therapies in the future [68].

Also, in the field of tissue engineering, SMPs have emerged as a transformative tool. SMP-based scaffolds offer a remarkable ability to mimic the mechanical properties of natural tissues, making them invaluable for repairing or replacing damaged organs and structures. These scaffolds can be custom-designed to match specific tissue types, providing an ideal environment for cells to grow, differentiate, and regenerate. Whether it is recreating the elasticity of muscle tissue or the rigidity of bone, SMPs enable precise control over the mechanical characteristics of the scaffold. Moreover, SMPs can respond to external stimuli, such as temperature changes, enabling dynamic adjustments to better accommodate tissue growth and healing [69]. This innovation in tissue engineering holds immense potential in addressing a wide range of medical challenges, from organ transplantation to the repair of damaged cartilage and bone, ultimately improving patients’ quality of life and advancing the field of regenerative medicine [70].

What is more, in the orthopedics sector, SMPs have introduced groundbreaking advancements in the design and performance of orthopedic implants. These smart materials are particularly beneficial for bone plates, screws, and joint replacements. SMP-based implants offer a remarkable combination of properties, including improved stability and patient comfort. When used in bone plates and screws, SMPs adapt to the surrounding bone tissue, reducing the risk of implant failure and enhancing long-term durability. This adaptability also minimizes stress on adjacent tissues, leading to improved patient comfort and reduced postoperative pain. In the context of joint replacements, SMPs allow for a more natural range of motion by closely matching the mechanical properties of the surrounding bone and cartilage. Furthermore, they can be engineered to gradually degrade over time, eliminating the need for surgical removal and reducing the risk of complications. These advancements in orthopedic implants underscore the potential of SMPs to enhance the quality of life for individuals with musculoskeletal conditions and injuries [71].

Likewise, in the field of vascular medicine, SMPs have revolutionized the development of stents and other vascular devices, offering safer and more effective treatment options. SMP-based vascular devices are designed to respond to specific external stimuli, typically temperature, enabling them to adapt and expand precisely within blood vessels. This property is particularly advantageous during minimally invasive procedures such as angioplasty and stent placement [72]. SMPs allow these devices to be introduced into the vascular system in a compact state, reducing the risk of damage during insertion. Once inside the blood vessel, they respond to the body’s temperature, expanding and providing necessary support to keep the vessel open and restore healthy blood flow. This capability minimizes complications, such as vessel injury and restenosis, while improving the long-term effectiveness of vascular interventions. Shape memory polymers have undoubtedly elevated the standards of care in vascular medicine, offering patients safer and more efficient solutions for various cardiovascular conditions [72].

**Figure 9:**
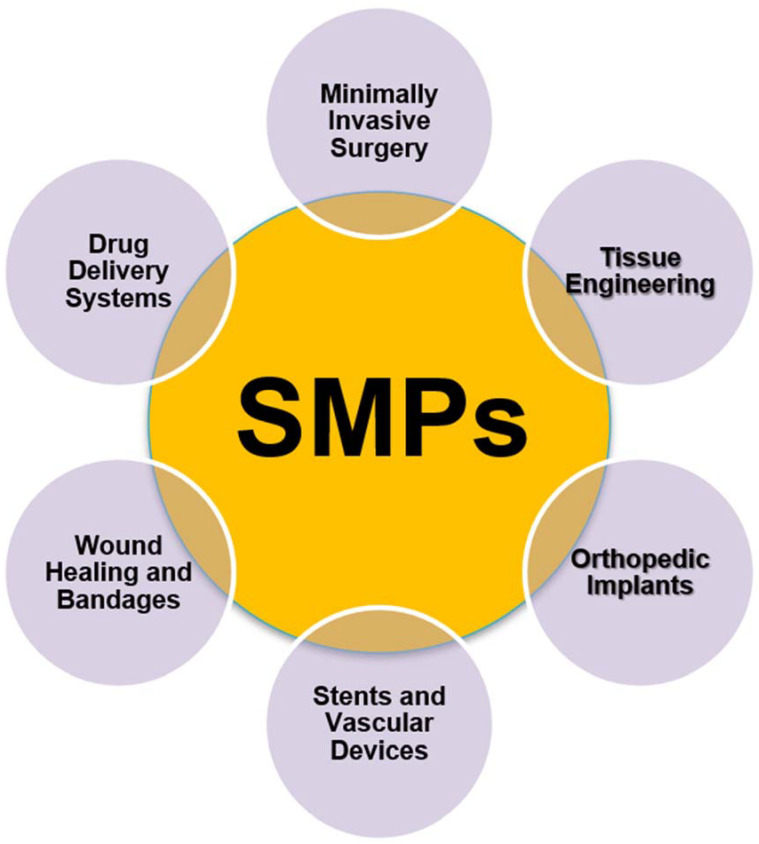
Applications of shape memory polymers (SMPs) in the biomedical field.

Moreover, regarding wound healing and medical bandages, shape memory polymers (SMPs) have emerged as a cutting-edge solution that combines adaptability and therapeutic efficacy. SMP-based bandages have been engineered to conform seamlessly to the unique contours of wounds and injured areas on the body. This conformability not only enhances patient comfort but also promotes optimal wound contact, which is crucial for effective healing. These bandages can be designed to respond to various stimuli, such as temperature or moisture levels, to provide tailored wound care. Furthermore, some SMP-based bandages are capable of releasing medications or growth factors in response to specific triggers, thereby facilitating the healing process by delivering therapeutic agents directly to the wound site [73]. This innovation represents a significant step forward in wound care, as it not only accelerates the healing process but also minimizes the risk of infection and scarring, ultimately improving the overall quality of patient care. As technology and research continue to advance, SMP-based bandages hold promise for addressing various wound types and complexities, offering patients a more efficient and comfortable path to recovery. Figure 9 depicts the applications of shape memory polymers (SMPs) in the biomedical field [74].

### 3.2. LCEs Applications

LCEs have emerged as a groundbreaking category of materials that, when incorporated with 4D printing technology, have the potential to revolutionize the field of regenerative medicine. Smart materials, renowned for their distinctive reaction to external stimuli, present a wide range of applications that are reshaping the fields of tissue engineering, regeneration, and patient-centered care [75].

LCEs have revolutionized the sector of tissue scaffolds within the field of regenerative medicine. These materials exhibit a distinctive amalgamation of flexibility and responsiveness, rendering them well-suited for the fabrication of dynamic scaffolds capable of adapting and evolving in real-time with regenerating tissue. Scaffolds based on liquid crystal elastomers (LCEs) exhibit regulated form changes in response to external stimuli, such variations in temperature or exposure to light. The observed dynamic activity exhibits a remarkable resemblance to the contractile characteristics of native tissue, therefore offering a significant and innovative prospect in areas such as cardiac tissue engineering [75].

In the field of cardiac regenerative medicine, LCE-based scaffolds have the ability to replicate the dynamic mechanical properties shown by the heart. The cardiac cells possess the ability to undergo rhythmic expansion and contraction, which is in direct response to the electrical impulses generated by the heart. This physiological characteristic enables these cells to create an environment that closely mimics the properties of original tissue. Dynamic mimicry has several advantages. It facilitates the synchronization and development of heart cells, resulting in enhanced functioning. Moreover, it facilitates the enhancement of contractile power in the designed tissue, a critical factor in ensuring successful heart regeneration [76].

Additionally, scaffolds based on LCEs have the capability to be programmed in a manner that allows them to selectively respond to particular stimuli present inside the biological system. For example, these organisms possess the ability to detect alterations in pH levels or the existence of certain proteins that are linked to the process of tissue regeneration. In response, these scaffolds have the capability to release growth factors or signaling molecules with spatial and temporal precision, therefore augmenting the regeneration process [76].

LCE-based scaffolds have the capability to be deliberately designed in a manner that enables them to react to certain external stimuli or circumstances present inside the biological system. For example, these sensors can be engineered to exhibit sensitivity towards variations in temperature, pH levels, or the existence of particular biochemical indicators linked to the process of tissue regeneration. When the aforementioned triggers are identified, liquid crystal elastomers (LCEs) have the ability to undergo alterations in their form, release bioactive compounds, or adjust their mechanical characteristics in a dynamic manner [77].

From a practical standpoint, it can be observed that constructions based on LCE have the capability to selectively release growth factors, signaling molecules, or other bioactive substances at the specific time and location required for tissue repair and regeneration. In the context of bone tissue engineering, it has been observed that LCE scaffolds possess the ability to detect changes in pH levels during the process of bone fracture repair. When the pH level indicates a requirement for increased mineralization, the LCE scaffold has the capability to release growth factors that promote bone formation, therefore expediting the process of bone regeneration [77].

In addition, it should be noted that LCEs have the ability to replicate the mechanical stimuli that naturally occur in the microenvironment of tissues. In the context of muscle tissue regeneration, it is possible to utilize LCE-based scaffolds that possess the ability to adapt their stiffness and elasticity in response to mechanical pressures. The aforementioned dynamic behavior plays a crucial role in directing the alignment and maturation of muscle cells, ultimately culminating in the development of fully functioning muscular tissue [78].

The utilization of LCE-based constructs in regenerative medicine has revolutionized the field by enabling precise control over cell signaling and the tissue microenvironment. This technology facilitates the ability of researchers and doctors to precisely adjust the regeneration process, hence enhancing the ideal cellular response during every phase of tissue healing. The degree of control achieved in this context not only amplifies the efficacy of regenerative therapies but also facilitates the exploration of customized and individualized treatments, therefore enhancing the overall well-being of patients undergoing regenerative operations [78].

The integration of LCEs with 4D printing technology presents an opportunity in the development of personalized implants, which are designed to cater to the distinct anatomical and physiological attributes of individual patients. This application signifies a notable transformation in the field of regenerative medicine, as the conventional approach of employing universal remedies is being replaced by individualized and precise therapies. Through the utilization of the design adaptability inherent in liquid crystal elastomers (LCEs), medical practitioners have the capability to fabricate implants that precisely align with the individual requirements of a certain patient. The initial step in this procedure is the collection of patient data, encompassing medical imaging, genetic information, and pertinent clinical indicators. Based on the available data, it is possible to create LCE-based implants with a high level of precision, allowing for a remarkable replication of the patient’s anatomical structure.

In the field of orthopedics, which frequently involves joint replacement surgery, LCEs may be utilized to manufacture joint implants that are tailored to the unique needs of individual patients. The implants have the capability to accurately replicate the contour, dimensions, and mechanical characteristics of the individual’s joint, hence guaranteeing an optimal alignment. This not only improves the compatibility of implants but also promotes their performance, hence decreasing the likelihood of difficulties after surgery and enhancing the patient’s overall quality of life [79].

LCE-based implants have been utilized in craniofacial surgery to achieve facial bone reconstruction, providing enhanced cosmetic and functional outcomes as compared to conventional implants. The accurate replication of a patient’s face structure represents a significant advancement in bolstering the patient’s self-assurance and general state of health. Moreover, it is possible to design LCEs in such a way that they exhibit a targeted response to particular environmental stimuli present in the human body. For example, bacteria have the ability to adjust to variations in temperature or pH levels, so increasing their compatibility with the physiological conditions of the patient. The implant’s capacity to react dynamically guarantees that it maintains both comfort and functionality among the patient’s normal bodily variations [80].

Also, the utilization of LCEs in additive manufacturing allows for highly controlled deterioration rates, a much needed characteristic within the domain of regenerative medicine. This innovative method holds promise in mitigating the necessity for subsequent surgical interventions. The present application effectively tackles a significant issue within the sector by offering a solution that improves patient comfort, reduces healthcare expenses, and mitigates the potential risks associated with supplementary treatments [80].

The structures based on LCE can be intentionally designed to undergo slow degradation over a period of time, in order to synchronize with the rate of tissue regeneration. The controlled disintegration of the scaffold or implant is meticulously managed to maintain its structural integrity and functionality during the crucial first phases of tissue regeneration. The LCE scaffold undergoes spontaneous degradation when newly formed tissue undergoes maturation, obviating the necessity for intrusive removal treatments [81].

LCE-based structures provide comparable benefits in soft tissue regeneration, particularly in the context of repairing cartilage injury. The scaffold plays a crucial role in offering primary mechanical assistance to the regenerating tissue while also accommodating the dynamic requirements of the healing process. As the tissue undergoes the healing process and attains self-sustaining properties, the LCE scaffold undergoes a slow degradation, so enabling the patient to restore the inherent functionality of the joint without necessitating further surgical procedures [81].

The implementation of this regulated degrading process represents a noteworthy advancement in the development of regenerative medicines that are both more accommodating to patients and more economically viable. This is in accordance with the philosophy of reducing patient discomfort and healthcare burdens, hence improving the entire patient experience.

In addition, LCEs demonstrate remarkable biocompatibility. The purpose of these materials is to effectively engage with the biological processes present in the human body, facilitating cellular adhesion, proliferation, and aiding the regeneration of tissues. Thus, it is possible to manipulate liquid crystal elastomers (LCEs) in order to mitigate the adverse effects of toxicity and inflammation, both of which are frequently encountered when incorporating foreign substances into the human body. LCE-based structures are intentionally engineered to possess inherent biocompatibility, hence mitigating the potential for unfavorable responses and assuring their favorable acceptance by the host tissue [82].

Furthermore, it should be noted that LCEs possess a distinctive benefit in their capacity to facilitate the integration of tissues. When employed as scaffolds or implants, these materials offer a conducive milieu for cellular colonization and functionality. LCE-based constructs have the capability to be deliberately designed with surface features that exhibit an enhanced affinity for cell adhesion, hence facilitating a robust attachment of cells to the scaffold. The first attachment is a crucial stage in the process of tissue regeneration since it establishes the fundamental basis for the subsequent development of new tissue.

Moreover, it has been observed that LCEs have the ability to replicate the mechanical characteristics of natural tissue. LCE-based scaffolds have the potential to be customized in musculoskeletal applications, namely in ligament or tendon restoration, in order to align with the mechanical characteristics of the adjacent tissue. This guarantees that the scaffold offers the requisite degrees of support and signals for cells to align and develop, ultimately resulting in the regeneration of functional tissue.

**Figure 10:**
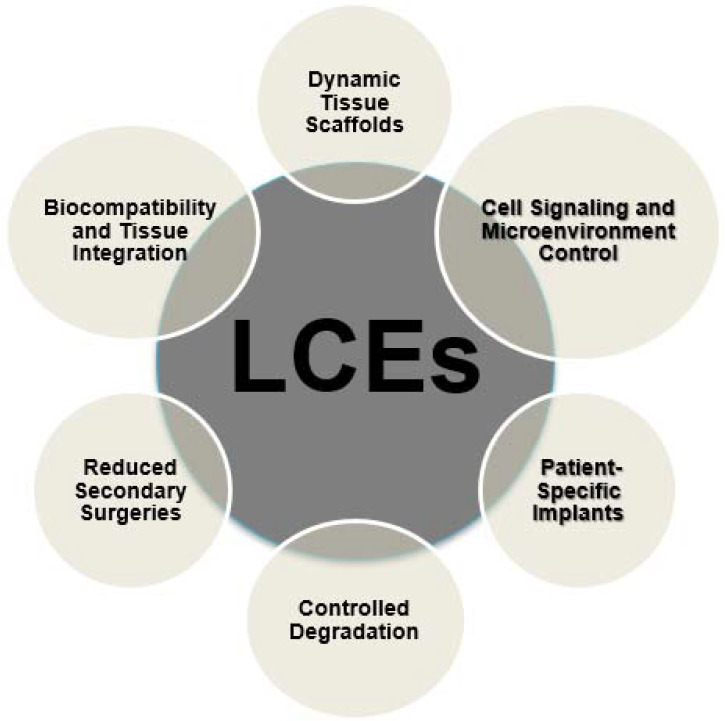
Applications of LCEs in the biomedical field.

Additionally, it is noteworthy that liquid crystal elastomers (LCEs) possess the capability to react to mechanical stimuli, a characteristic that holds significant relevance in several fields, including the domain of cardiac tissue engineering. The dynamic characteristics of scaffolds based on liquid crystal elastomers (LCEs) have the ability to conform to the contractile properties of the heart, hence facilitating the alignment and maturation of cardiac cells. The alignment of the regenerated tissue contributes to the enhancement of its functional qualities, leading to enhanced contractility and overall cardiac performance. Figure 10 depicts the applications of LCEs in the biomedical field [83].

### 3.3. Magnetic Shape Memory Alloys (MSMAs) Applications

MSMAs have the potential to significantly transform the field of regenerative medicine through its integration with 4D printing technology. The aforementioned materials, renowned for their capacity to undergo form alterations when subjected to magnetic fields, are presenting promising prospects in the sectors of tissue engineering, regeneration, and patient-centered healthcare [84].

MSMAs offer an unprecedented capability to create dynamic tissue engineering constructs. By leveraging their responsiveness to magnetic fields, MSMA-based scaffolds and implants can be tailored to adapt and evolve in real-time within the body. In applications such as musculoskeletal regeneration, MSMA-based implants can mimic the dynamic mechanical properties of native tissue. They can respond to the patient’s movements, providing the necessary support and cues for cells to align and mature. This dynamic behavior accelerates tissue repair and enhances functional tissue regeneration [84].

MSMAs enable precise control over cellular responses through the application of magnetic fields. Magnetic cues can guide cell migration, alignment, and differentiation within MSMA-based constructs. For instance, in neural tissue engineering, MSMA-based scaffolds can be used to guide the direction of axonal growth in spinal cord repair. Magnetic fields can be applied to manipulate the orientation of the MSMAs, subsequently directing the growth of neural cells and fostering the reconnection of damaged neural pathways.

One of the most transformative applications of MSMAs in 4D printing is the creation of patient-specific implants. By incorporating patient data, including medical imaging and anatomical details, into the design process, clinicians can fabricate MSMA-based implants that precisely match the patient’s unique requirements. This level of personalization not only enhances implant compatibility but also optimizes functionality and reduces the risk of implant rejection. For instance, in joint replacement surgeries, MSMA-based implants can be customized to fit the patient’s exact joint dimensions, promoting a more natural range of motion and improved patient outcomes [85].

**Figure 11:**
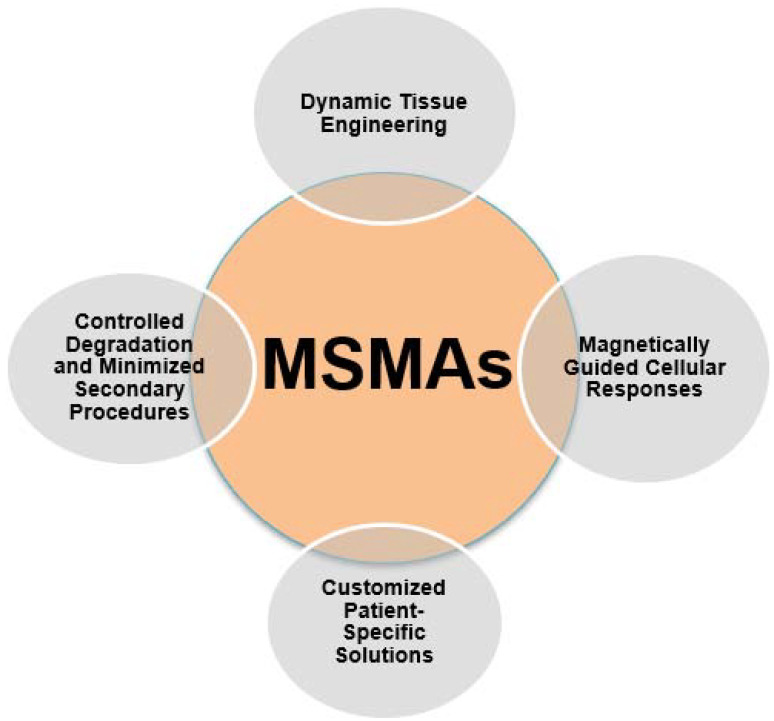
Applications of magnetic shape memory alloys (MSMAs) in the biomedical field.

MSMA-based constructs can be designed to degrade gradually over time, in response to magnetic fields. This controlled degradation aligns with the pace of tissue regeneration, eliminating the need for secondary surgeries to remove implants or scaffolds. As new tissue forms and matures, the MSMA-based construct naturally transforms and ultimately dissolves, reducing patient discomfort, healthcare costs, and the risk of complications associated with additional interventions. This patient-friendly approach enhances the overall regenerative experience. Figure 11 depicts the applications of magnetic shape memory alloys (MSMAs) in the biomedical field.

### 3.4. Biodegradable Polymers Applications

Biodegradable polymers have become recognized as a sustainable and adaptable category of materials, with significant implications for regenerative medicine when included into 4D printing technology. The utilization of these polymers, which are engineered to undergo natural degradation, presents a multitude of applications that are significantly transforming the fields of tissue engineering, regenerative therapies, and patient-centric healthcare.

They enable the creation of temporary support structures in regenerative medicine. In 4D printing, these materials can be precisely shaped into scaffolds or implants that provide initial structural support to regenerating tissue. As the tissue heals and matures, the biodegradable scaffolds gradually break down, eliminating the need for invasive secondary surgeries to remove permanent implants. This patient-friendly approach minimizes discomfort, reduces healthcare costs, and lowers the risk of complications, setting a new standard for regenerative therapies [86].

Additionally, biodegradable polymers have found a crucial role as drug delivery systems in regenerative medicine. In 4D printing, these polymers can be formulated to encapsulate bioactive agents, such as growth factors or signaling molecules. As the biodegradable construct degrades over time, it releases these agents in a controlled and targeted manner. For instance, in wound healing, biodegradable dressings can be 4D-printed to deliver growth factors at specific stages of tissue repair, promoting faster healing and reducing scarring.

Their use in 4D printing allows for the fabrication of scaffolds that gradually degrade as new tissue forms. These scaffolds mimic the natural extracellular matrix, providing a supportive environment for cell attachment and tissue growth. For instance, in cartilage regeneration, biodegradable scaffolds can be 4D-printed to match the mechanical properties of native cartilage and degrade over time as the regenerated tissue matures. This approach eliminates the need for scaffold removal procedures, simplifying the regenerative process [86].

**Figure 12:**
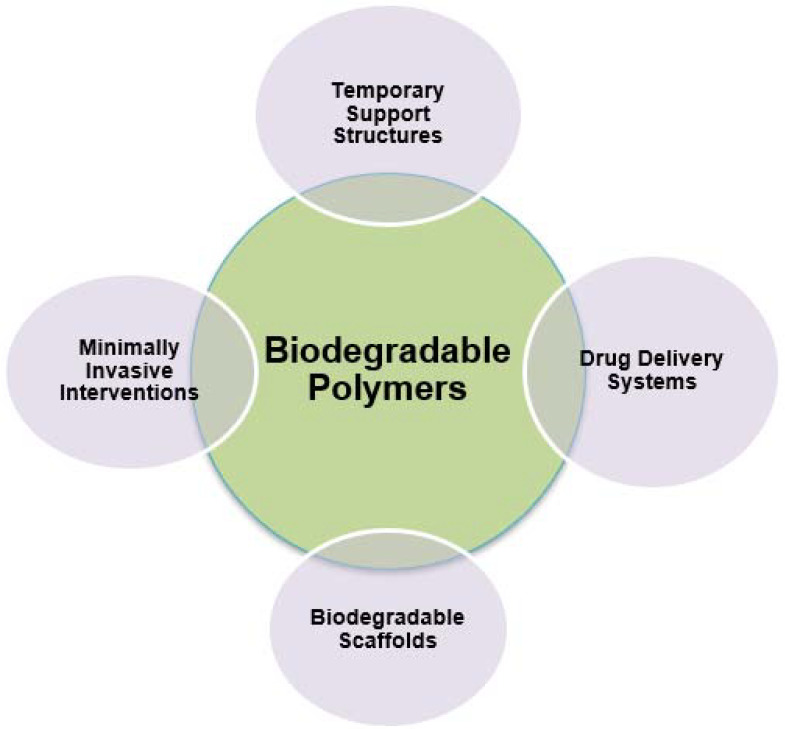
Applications of biodegradable polymers in the biomedical field.

Lastly, biodegradable polymers are instrumental in advancing minimally invasive interventions in regenerative medicine. Constructs made from these polymers can be 4D-printed to be compact for minimally invasive delivery, then expand to their functional shape once in place. This eliminates the need for more invasive open surgeries, reducing patient trauma and recovery times. For instance, biodegradable stents can be delivered minimally invasively and gradually degrade as the blood vessel heals and regenerates [87]. Figure 12 depicts the applications of biodegradable polymers in the biomedical field.

### 3.5. Cell-Laden Materials Applications

The incorporation of cell-laden substances into 4D printing has introduced a novel phase in the field of regenerative medicine, facilitating the development of dynamic, living structures capable of adapting and evolving throughout their lifespan. These materials have distinct characteristics that render them highly attractive for applications in tissue engineering and regenerative medicine [88].

Cell-laden materials empower the development of personalized tissue constructs. Through the use of patient-specific data, including medical imaging and genetic information, these materials can be precisely tailored to replicate an individual’s unique anatomical features. In 4D printing, cell-laden constructs are engineered to mimic the complexity of native tissues, promoting optimal compatibility and functionality. For example, in organ transplantation, these constructs offer the potential to create patient-specific, functional organs that minimize the risk of rejection and improve overall transplant outcomes [88].

Furthermore, they provide a platform for controlling cellular microenvironments with unparalleled precision. Researchers and clinicians can manipulate the composition, spatial arrangement, and biochemical cues within these constructs to guide cell behavior. This control is invaluable for orchestrating tissue regeneration. For instance, in neural tissue engineering, cell-laden materials can be designed to release neurotrophic factors that encourage nerve cell growth and connections, aiding in spinal cord injury repair [89].

The dynamic nature of 4D printing allows cell-laden materials to adapt and evolve with changing tissue needs. These constructs can be programmed to respond to specific cues within the body, adjusting their properties over time. For example, in cardiac tissue engineering, cell-laden scaffolds can mimic the contractile behavior of the heart by altering their mechanical properties in response to electrical signals. This adaptability promotes the maturation of cardiac cells and improves the functional integration of the regenerated tissue [90].

Moreover, they offer a sophisticated platform for controlled drug delivery. By embedding therapeutic agents within the constructs, researchers can precisely modulate the release of bioactive molecules, growth factors, or medications. This feature allows for the temporal and spatial regulation of cellular behavior during tissue repair and regeneration. For instance, in bone regeneration, cell-laden scaffolds can release bone growth factors at specific stages of the healing process, accelerating bone formation [91].

**Figure 13:**
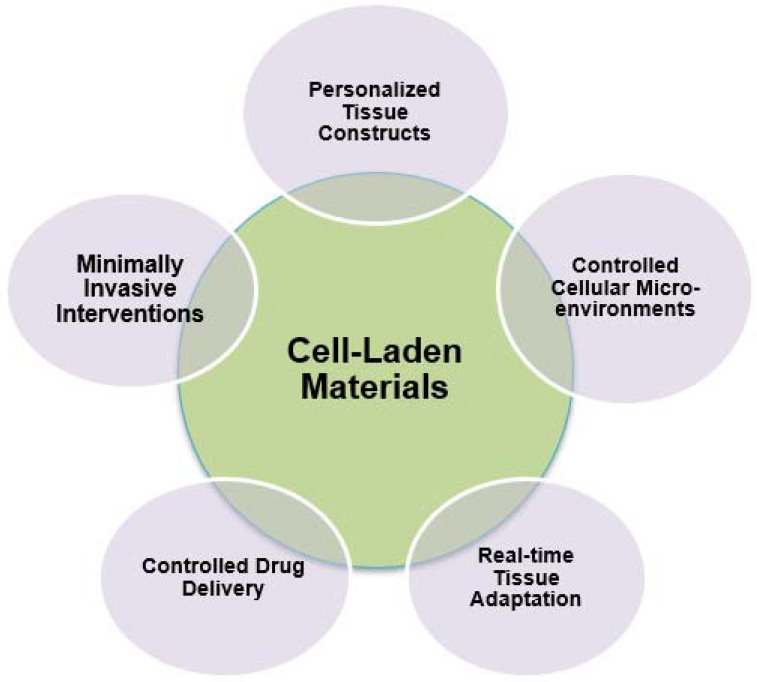
Applications of cell-laden materials in the biomedical field.

What is more, cell-laden materials in 4D printing contribute to the advancement of minimally invasive interventions in regenerative medicine. These constructs can be designed to take on a specific shape or function once inside the body, reducing the invasiveness of procedures. For example, cell-laden stents can be compressed for minimally invasive delivery and then expand to their functional shape once in place, providing support to the regenerating tissue without the need for open surgery. In essence, the utilization of cell-laden materials in the context of 4D printing holds significant potential for revolutionizing the field of regenerative medicine. The capacity to generate customized tissue constructions, manipulate cellular microenvironments, dynamically adjust to changing conditions, ease precise drug administration, and support less invasive interventions is profoundly influencing the trajectory of regenerative treatments. Figure 13 depicts the applications of cell-laden materials in the biomedical field [91].

## 4. Discussion

The incorporation of intelligent materials such as SMPs, LCEs, MSMAs, biodegradable polymers, and cell-laden materials in the field of additive manufacturing (AM), specifically in the domain of regenerative medicine, presents significant opportunities for transforming healthcare and tissue engineering [92]. Table 1 lists the aforementioned materials, their applications, and their most prominent features such as strength, cost-effectiveness, biocompatibility, etc.

Nevertheless, the adoption of these technologies also presents a multitude of notable obstacles that must be effectively handled in order to ensure successful implementation.

One of the foremost challenges in incorporating the aforementioned materials in this field is ensuring their compatibility with biological systems and their ability to foster the regenerative process. These materials need to seamlessly integrate with living tissues and cells without causing adverse reactions, inflammation, or toxicity. Achieving this level of compatibility requires a deep understanding of the intricate interactions between the materials and biological environments. Moreover, the biofunctionality of these materials plays a pivotal role [93]. They should not merely coexist with biological entities but actively support and promote cellular growth, differentiation, and tissue regeneration. Designing materials that mimic the extracellular matrix, which serves as a natural scaffold for cells, is a complex undertaking. Researchers must meticulously engineer smart materials to encourage cell adhesion, proliferation, and the production of necessary extracellular matrix components like collagen and elastin. Furthermore, smart materials should respond appropriately to physiological cues, such as changes in pH, temperature, or mechanical forces, to facilitate tissue development and adaptation. Achieving this level of biofunctionality necessitates multidisciplinary collaboration between materials scientists, biologists, and biomedical engineers. Rigorous testing and validation are essential to assess the safety and efficacy of these materials in vivo, ensuring that they not only support the regenerative process but also maintain their functionality over time [94].

The stability of smart materials throughout the intricate process of additive manufacturing (AM), especially when employed for regenerative medicine applications, presents a multifaceted challenge. These materials often exhibit sensitivity to environmental factors such as temperature, humidity, and pH levels. During the printing process, maintaining the structural and functional integrity of smart materials becomes paramount. Variations in ambient conditions can lead to unintended changes in material properties, jeopardizing the precision and reliability of the printed constructs. To address this challenge, researchers and engineers must develop specialized printing methodologies that carefully control environmental conditions within the printing chamber [95]. This may involve the use of controlled atmospheres, temperature regulation, and humidity management systems. Additionally, post-processing steps may be required to stabilize printed structures further. These steps might include curing, cross-linking, or annealing processes tailored to the specific smart material used. Furthermore, variations in material composition or impurities can also impact stability, emphasizing the importance of quality control throughout the manufacturing process. Ensuring uniformity in material properties and structural integrity can be particularly challenging when dealing with intricate 4D printing, where materials may undergo shape or property changes over time. Thus, maintaining material stability during printing and post-processing stages remains a crucial challenge in harnessing the potential of smart materials for AM in regenerative medicine, necessitating continuous research and development efforts [96].

Another compelling, yet intricate challenge arising, is the necessity for advanced and sophisticated printing techniques. Unlike traditional 3D printing, 4D printing involves materials that can dynamically change shape, properties, or functions over time in response to external stimuli. This added dimension of temporal transformation requires a higher level of control and precision during the printing process. To successfully address this challenge, researchers and engineers must develop and employ specialized printing equipment capable of orchestrating the precise temporal and spatial changes in smart materials. This may involve multi-material printing setups, where different materials are deposited sequentially or simultaneously, or it may require the integration of responsive elements like temperature controllers or magnetic fields to trigger the desired transformations [97]. Consequently, 4D printing necessitates a deep understanding of material behaviors, programming algorithms, and the intricacies of the intended shape or property changes. Moreover, achieving reproducibility and reliability in 4D-printed constructs is a complex endeavor. The fine-tuning of printing parameters, including material deposition rates, temperatures, and environmental conditions, is critical to ensuring that the final product functions as intended. Furthermore, researchers must develop user-friendly design and simulation tools that allow for the precise specification of desired transformations, making 4D printing accessible to a broader range of applications in regenerative medicine field [98].

Other potential drawbacks can be both elevated material costs and rigorous regulatory approval. These materials, often requiring complex synthesis and specialized processing techniques, can be prohibitively expensive, with MSMAs, for instance, involving intricate alloy compositions. These high material costs can potentially hinder the scalability and affordability of 4D printing applications in the field of regenerative medicine. Simultaneously, navigating the regulatory landscape for these advanced materials can be a formidable task [99]. They fall under the category of advanced therapies, subjecting them to strict regulatory scrutiny, which is time-consuming and costly. Researchers and companies must collaborate closely with regulatory agencies to establish clear pathways for approval, conduct thorough preclinical testing, and demonstrate both safety and efficacy in clinical trials. Effectively addressing these dual challenges is imperative to ensure that advanced AM approaches with smart materials become financially viable and can ultimately deliver safe and effective regenerative therapies to patients worldwide [100].

Also, the seamless integration of smart materials into such applications demands not only rigorous adherence to regulatory standards but also a comprehensive assessment of long-term performance and durability. The complex nature of these materials, their intricate interactions with biological systems, and the novelty of 4D printing technologies pose unique challenges in navigating the regulatory approval process. Researchers and companies must engage closely with regulatory agencies to establish clear pathways for approval, conduct robust preclinical testing, and document material properties, biocompatibility, and safety profiles. This process is not only time-consuming but also costly. Simultaneously, assessing the long-term performance and durability of these materials in a biological environment presents its own set of challenges. Predicting how smart materials will behave over extended periods, including potential degradation or loss of functionality, requires extensive testing and validation. Ensuring compliance with safety and efficacy standards is paramount for both regulatory approval and the sustained success of these innovative materials in regenerative therapies. It necessitates a holistic approach that combines scientific rigor, clinical evidence, and regulatory diligence to ensure the highest levels of patient safety and treatment effectiveness [100].

What is more, the successful utilization of these materials requires expertise spanning materials science, biology, engineering, and medicine. Bridging the gap between these diverse disciplines is essential for progress. Effective communication and collaboration between researchers, engineers, and healthcare professionals are vital to develop innovative solutions and translate them into practical clinical applications. Moreover, this endeavor necessitates a skilled workforce capable of working with these cutting-edge materials and advanced AM techniques. Establishing comprehensive education and training programs is imperative to equip researchers and practitioners with the necessary knowledge and skills. This includes providing hands-on training in materials engineering, 4D printing technologies, and an understanding of the unique challenges posed by smart materials in the context of regenerative medicine. By fostering collaboration and education, we can maximize the transformative potential of smart materials in the field while ensuring that the expertise required to work with them is readily available and continually evolving [101].

Lastly, a host of ethical considerations that demand comprehensive examination are brought forth. Beyond the scientific and technical aspects, it is imperative to delve into the ethical dimensions of this innovation. Central to these concerns are issues of informed consent, privacy, and equitable access to advanced treatments. Informed consent takes on new dimensions as patients engage with cutting-edge therapies involving smart materials [102]. Ensuring that patients fully understand the implications, potential risks, and benefits of these novel treatments is of utmost importance. Privacy concerns arise as the use of smart materials often involves the collection of sensitive health data, necessitating stringent data protection and patient confidentiality measures. Additionally, addressing disparities in access to these groundbreaking treatments is essential to prevent furthering existing healthcare inequalities. Ethical discourse must play a pivotal role in guiding the responsible development, dissemination, and utilization of smart materials in regenerative medicine to ensure that ethical principles and patient rights are upheld at every stage of implementation [103].

## 5. Conclusions

The evolving landscape of regenerative medicine has introduced dynamic and biomimetic solutions, thanks to the integration of smart materials within additive manufacturing processes. This manuscript underscores the transformative role of these materials, which can adapt to biological cues and environmental changes, thereby revolutionizing tissue engineering.

By showcasing the innovative fusion of shape memory polymers and stimulus-responsive hydrogels in additive manufacturing, this research highlighted the unprecedented potential to engineer tissue constructs that closely mimic natural tissue behaviors. Notably, the ability to create dynamically adaptable scaffolds and responsive drug delivery systems holds immense promise for personalized and effective regenerative therapies.

This study accentuates the forward-looking nature of regenerative medicine, where smart materials serve as pioneers in shaping the field’s future. It emphasizes the necessity of addressing material compatibility, stability, regulatory considerations, and the imperative need for interdisciplinary collaboration. These findings herald a future where patients can benefit from advanced treatments, fostering optimism, healing, and a brighter healthcare outlook.

The manuscript’s key contribution lies in its demonstration of how smart materials are redefining the boundaries of tissue engineering, signaling a paradigm shift from static constructs to responsive, biomimetic solutions. This highlights the potential for further exploration and development, emphasizing the importance of overcoming current challenges to unlock the full potential of smart materials in regenerative medicine.

## Figures and Tables

**Table 1:** Smart materials, their applications, and their most prominent features.

Material	Applications	Prominent Features	References
Shape memory polymers (SMPs)	Tissue scaffolds, drug delivery systems, smart textiles, minimally invasive devices, soft actuators	Biocompatibility, stimulus-responsiveness, shape memory effect, tunable mechanical properties, 3D printability	[66,67,68,69,70,71,72,74]
Liquid crystal elastomers (LCEs)	Soft robotics, adaptive optics, biomedical devices, responsive materials, optical devices, photonics	Large actuation strains, optical properties, tunable response, shape-morphing capabilities, high sensitivity	[75,76,77,78,79,80,81,82]
Magnetic shape memory alloys (MSMAs)	Biomedical devices, microactuators, sensors, actuators, reconfigurable structures, magnetic resonance imaging (MRI)	Magnetic responsiveness, high precision, reversible shape change, precise control in small-scale applications, magnetic resonance compatibility	[48,49,50,51,52,53,84,85]
Biodegradable polymers	Tissue engineering, drug delivery, sustainable packaging, agricultural applications, 3D printing filaments	Eco-friendliness, controlled degradation, biocompatibility, mechanical versatility, versatile degradation rates, compostability	[15,16,29,41,54,55,56,57]
Cell-laden materials	Tissue engineering, organoids, disease modeling, personalized medicine, high-throughput screening	Cellular integration, biomimetic environments, physiological relevance, cellular differentiation, disease modeling capabilities, high-throughput compatibility	[13,14,58,61,63,64,88]
